# Brown syndrome in one pair of dizygotic twins: a case report

**DOI:** 10.1186/1757-1626-3-1

**Published:** 2010-01-02

**Authors:** Abbas Attarzadeh, Abbas Hoseinirad, Feisal Rahat

**Affiliations:** 1 Poostchi eye research center, shiraz university of medical sciences, shiraz, Iran

## Abstract

**Introduction:**

Brown syndrome is a rare ocular movement abnormality. This syndrome is characterized by an inability to elevate the affected eye in adduction. Most cases are sporadic but the occurrence in Monozygotic twins has suggested the possible autosomal dominant inheritance in Brown syndrome.

**Case presentation:**

A 4-year-old girl (one pair of dizygotic twins) was referred to our pediatric ophthalmology clinic to assess her abnormal eye movement noticed by her mother. Visual acuity of both eyes was 20/20 with Snellen chart. Ocular motility showed mild exotropia in primary position with marked divergence in upward gaze (V pattern), mild hypotropia in adduction, and limitation of elevation in adduction of both eyes (Fig. 1A). We also examined her sister, all ocular evaluations including visual acuity, slit lamp examination, funduscopy and ocular motility (Ductions & Versions) were normal without any limitation.

We also review the related articles that previously have reported Brown syndrome in twins.

**Conclusion:**

Although there are few case reports of Brown syndrome in twins, combination of these reports may elucidate the genetic basis of this disease.

## Introduction

In 1950 Brown described an eye movement anomaly as the superior oblique (SO) tendon sheath syndrome [1]. He suspected the existence of a congenitally short SO tendon sheath, which restricted passive elevation of the adducted eye [2]. Brown syndrome is a rare ocular movement abnormality and most cases are sporadic and unilateral, but may be bilateral in 10% of cases [3]. Familial occurrence has been reported by several authors [4]. Also there are few case reports of Brown syndrome in twins that might be indicative of a possible autosomal dominant or recessive inheritance. Several monozygotic twins with Brown syndrome have been reported, but we found only two reports of this syndrome in dizygotic twins[4,5]. Here we found bilateral Brown syndrome only in one pair of dizygotic twins.

## Case Report

**Figure 1:**
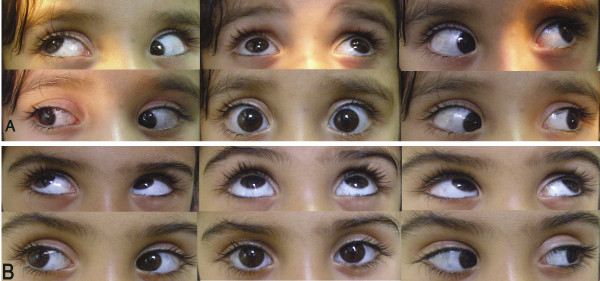
**A). Ocular movements of affected pair; note limitation of elevation in adduction of both eyes**. B) Normal eye motility of the other pair.

A 4-year-old girl (one pair of dizygotic twins) was referred to our pediatric ophthalmology clinic to assess her abnormal eye movement noticed by her mother. The patient had a history of 35 weeks gestational age at birth time and her birth weight was 1700 grams. Parents did not report any history of congenital abnormalities in the family. Pregnancy history was negative for exposure to drugs or teratogens. Visual acuity of both eyes was 20/20 with Snellen chart. Assessment of stereo acuity using the Titmus test wasn't possible. Cycloplegic refraction was +0.25 × 90° for both eyes. Ocular motility showed mild exotropia in primary position with marked divergence in upward gaze (V pattern), mild hypotropia in adduction, and limitation of elevation in adduction of both eyes (Fig. 1A). The patient had mild lid retraction in the primary position, but laboratory examination and consultation with relevant specialists was within normal limits. Fundus examination of both eyes was normal without any sign of objective torsion. All other examinations were within normal limits. We also examined her sister, all ocular evaluations including visual acuity, slit lamp examination, funduscopy and ocular motility (Ductions & Versions) were normal without any limitation. The past medical history of this pair was 1920 grams birth weight without any other problem.

## Discussion

Brown syndrome is caused by a mechanical restriction of the superior oblique tendon moving through the trochlea, which results in a restriction of elevation in adduction. Most cases of Brown syndrome are congenital, but it can be acquired due to either trauma or inflammation.

The occurrence of Brown syndrome, either bilateral or unilateral in one or both pairs of twins has been reported by several authors[4-9]. From these reports, only Magli et al. and Caldeira have reported ocular movement abnormality compatible with Brown syndrome in dizygotic twins, and other reports from Katz et al., Finaly et al., Lowe et al and kim et al. have been in monozygotic twins[5-7,9]. Table 1 summarizes these findings. Brown syndrome in reports of Finaly et al., Katz et al., kim et al. was equal in both pairs but Lowe RF^8 ^and Magli et al have found the Brown syndrome only in one pair of monozygotic and dizygotic twins, respectively[8]. In our report, we found that only one pair of dizygotic twins was affected and ocular motility of the other pair was without problem.

**Table 1:** Reported cases of brown syndrome in twins

Report	Affected Individuals	Gender	Laterality
**Lowe RF** **Magli et al**	Monozygotic Dizygotic	F M	Bilateral Unilateral
**Katz et al**	Monozygotic	F	Unilateral
**Finlay et al**	Monozygotic	F	Unilateral
**Kim et al**	Monozygotic	F	Bilateral
**Our report**	Dizygotic	F	Bilateral

Although all of the previous reports support a possible autosomal dominant or recessive inheritance with incomplete penetrance and variable expression, our case presentation like reports of Magli et al. and Caldeira strengthens the possibility of autosomal dominant with incomplete penetration or autosomal recessive transmission, because this syndrome was found only in one pair of twins and search for finding of similar disease in their siblings was negative[2].

Brown syndrome is a rare ocular motility abnormality and it seems if we merge these scattered case reports in twins, probable genetic basis as one of the ethiologic factors in Brown syndrome will be highlighted.

According to our search in the literature (MEDLINE from 1950 until 2008) we didnot find similar case report (bilateral Brown syndrome in one pair of dizygotic twins).

Our key words for search in MEDLINE were Brown syndrome with bilateral and twins.

Written informed consent was obtained from the patient for publication of this case report and accompanying images. A copy of the written consent is available for review by the Editor-in-Chief of this journal.

## Competing interests

The authors declare that they have no competing interests.

## Authors' contributions

AA participated in its design and coordination. AH wrote the article and provided the relevant material. FR done internet search and provided the patient photos.

All authors read and approved the final manuscript.

## References

[B1] Brown HW Allen JH Congenital structural muscle anomalies Strabismus Ophthalmic Symposium 1950 St. Louis: CV Mosby 205 36

[B2] Weakley DR Stager DR Stager DR Rosenbaum AL, Santiago AP "Brown syndrome" Clinical Strabismus Management 1999 Phliadelphia: WB Saunders 347 57

[B3] Wilson MM Eustis HS Jr Parks MM Brown's syndrome Surv Ophthalmol 1989 34 153 72 10.1016/0039-6257(89)90100-8 2694414

[B4] Caldeira JA Presumptive Brown's syndrome in dizygotic female twins: Case report and review of 30 familial cases in the literature Binocular Vision Strabismus Q 1996 11 23

[B5] Katz NK Whitmore PV Beauchamp GR Brown's syndrome in twins J Pediatr Ophthalmol Strabismus 1981 18 32 4 10.3928/0191-3913-19810101-09 7017099

[B6] Magli A Fusco R Chiosi E Del Bono G Inheritance of Brown's syndrome Ophthalmologica 1986 192 82 7 3703483 10.1159/000309618

[B7] Finlay A Powell S Brown's syndrome in identical twins Br Orthop J 1982 39 73 7

[B8] Lowe RF Bilateral superior oblique tendon sheath syndrome occurrence and spontaneous recovery in one of uniocular twin Br J Ophthalmol 1969 53 466 71 10.1136/bjo.53.7.466 5804031 PMC1207451

[B9] Kim SH Ben-Zion I Neely DE Bilateral Brown syndrome in monozygotic twins J AAPOS 2008 12 193 194 10.1016/j.jaapos.2007.10.009 18258471

[B10] Von Noorden GK Campos EC Binocular Vision and Ocular Motility 2002 6 St. Louis, Mosby 466 71

